# Contrast mimicking a subarachnoid hemorrhage after lumbar percutaneous epidural neuroplasty: a case report

**DOI:** 10.1186/1752-1947-7-88

**Published:** 2013-04-02

**Authors:** Chang Hyun Oh, Seong Dae An, Seung Hyun Choi, Gyu Yeul Ji

**Affiliations:** 1 Department of Neurosurgery, Guro Teun Teun Hospital, 1126-34, Guro 3-dong, Guro-gu, Seoul 152-880, Republic of Korea

## Abstract

**Introduction:**

Subarachnoid hemorrhage is one of the most feared acute neurologic events. Accurate diagnosis of subarachnoid hemorrhage is essential, and computed tomography of the brain is the first diagnostic imaging study. However, in rare circumstances, a similar appearance may occur in the absence of blood in the subarachnoid space. The contrast enhancement of subarachnoid space is a rare complication after lumbar percutaneous epidural neuroplasty, with, to the best of our knowledge, no previous report in the literature.

**Case presentation:**

A 42-year-old Korean male patient, who underwent a spinal operation five years previously at the level of L4 to S1, visited our clinic with persistent and aggravating low back pain. An imaging study revealed the focal and diffuse disc protrusion at the level of L4/5 and L5/S1. The clinician decided to perform a lumbar percutaneous epidural neuroplasty. During the procedure, dural adhesion was suspected at the previously operated level, and the neuroplasty catheter was malpositioned into the intradural space on the first attempt. After the catheter was repositioned, the scheduled epidural neuroplasty was completed. Our patient had no definite abnormal neurological signs. But, after a day, our patient complained of severe headache with sustained high blood pressure without neurological disorientation. Computed tomography of his brain showed a subarachnoid hemorrhage-like appearance with intracranial air. Sequential angiography, subtractional magnetic resonance imaging and examination of the cerebrospinal fluid revealed no abnormalities. Follow-up computed tomography after one day revealed no definite intracranial hemorrhage, and our patient was discharged with improved low back pain without neurological deficit.

**Conclusion:**

We report a rare case of contrast mimicking a subarachnoid hemorrhage after lumbar percutaneous epidural neuroplasty. The physician should keep in mind a rare case like this, and the supine position with head elevation is necessary to avoid a similar complication after lumbar percutaneous epidural neuroplasty.

## Introduction

Subarachnoid hemorrhage (SAH) is one of the most feared acute cerebrovascular events. Accurate diagnosis of SAH is essential because several diagnostic tests and therapies are indicated in the management of patients with SAH that are not routinely applied to patients with other acute neurologic events [1]. Cranial computed tomography (CT) is one of the first diagnostic imaging studies performed in suspected SAH. Although CT is less sensitive than magnetic resonance imaging (MRI), its sensitivity for SAH detection has been reported to be as high as 95% to 98% in patients scanned within 24 hours of symptom onset, and it is considered the study of choice for identification of SAH [2]. SAH appears on CT as hyperdensity in the subarachnoid space, a finding generally believed to be extremely specific [1]. However, in rare circumstances, a similar appearance may occur in the absence of blood in the subarachnoid space, a finding that has been termed pseudo, mimicking, or false positive SAH [1,3-8]. Contrast enhancement of subarachnoid space is a rare complication after lumbar percutaneous epidural neuroplasty (L-PEN). To the best of our knowledge, there have been no previous reports in the literature.

## Case presentation

**Figure 1:**
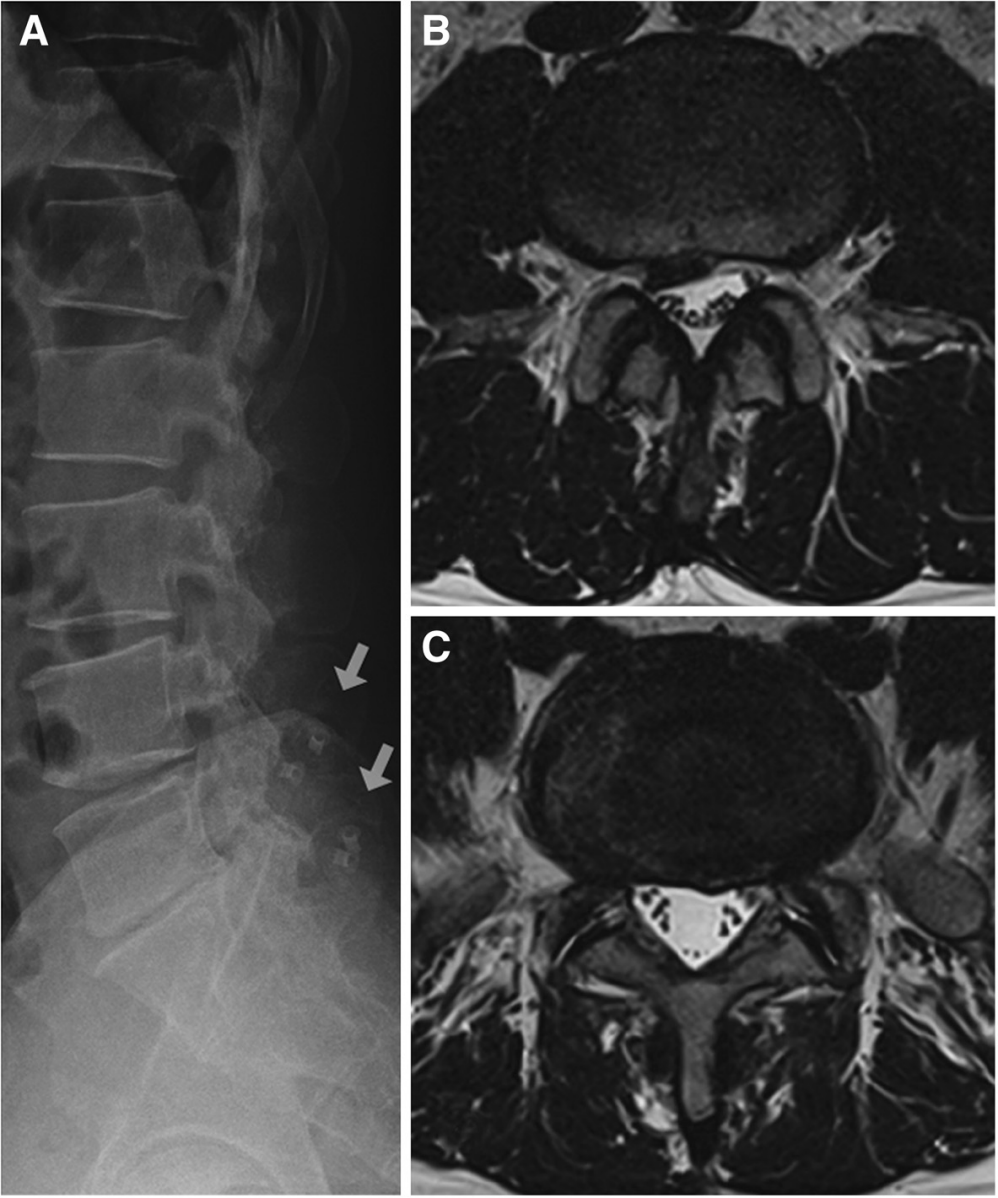
**Lumbar imaging studies. (A) **Simple lateral radiographs with the DIAM^®^ interspinous device (white arrows). **(B) **Focal disc protrusion at L4/5. **(C)** Diffuse protrusion at L5/S1.

A 42-year-old Korean male patient visited our clinic with persistent and aggravating low back pain. Five years ago he had a DIAM^®^ (Medtronic Sofamor Danek Inc., Minneapolis, MN, USA) interspinous process soft-stabilization system placed at the level of L4 to S1 (Figure 1). Current imaging revealed focal disc protrusion at the level of L4/5 and diffuse protrusion at the level of L5/S1 (Figure 1). We decided to perform an L-PEN at the lesion sites to relieve the sustained pain.

**Figure 2:**
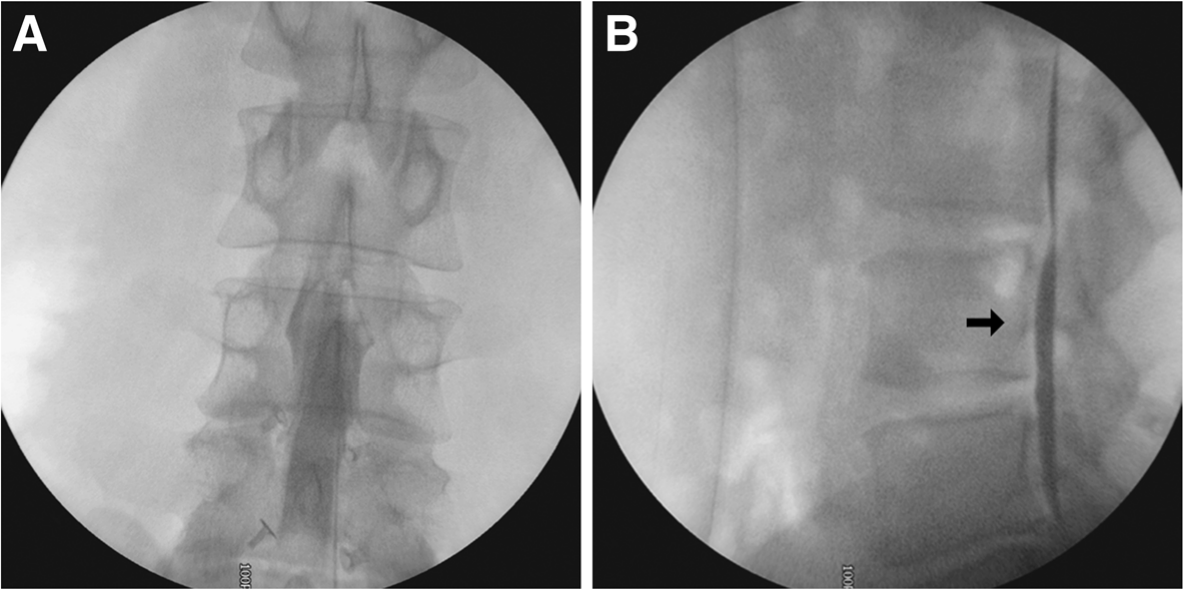
**Malpositioning of the neuroplasty catheter into the intradural space during the first attempt of lumbar percutaneous epidural neuroplasty because of dural adhesion at the previously operated sites. (A) **The myelogram-like contrast filling in the anteroposterior image and **(B) **the contrast band along the ventral side of the dura (black arrow).

During the procedure, dural adhesion was suspected at the previously operated level, and the neuroplasty catheter was malpositioned into the intradural space on the first attempt (Figure 2). After repositioning the epidural catheter and confirming its position, the scheduled epidural neuroplasty was completed. The total dose of contrast administered during the procedure was less than 3mL. Our patient had no definite abnormal neurological signs.

**Figure 3:**
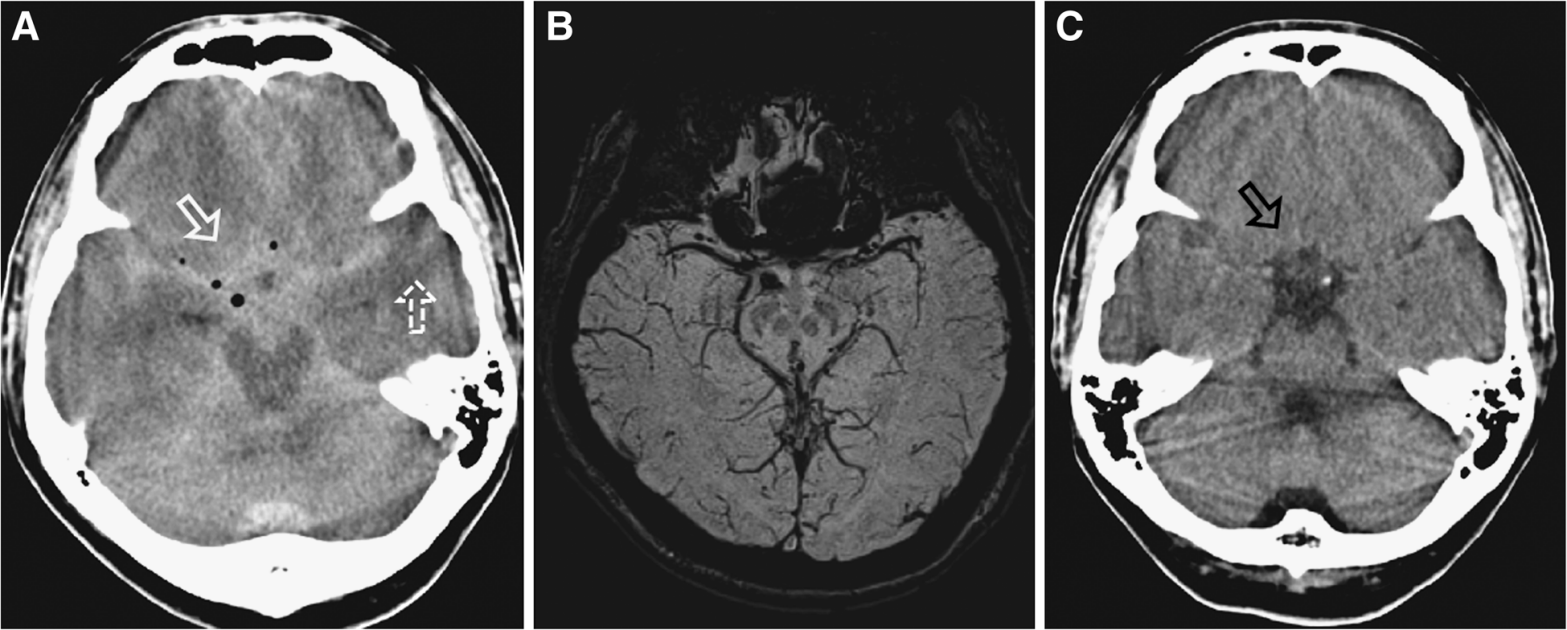
**Cranial images. (A) **The contrast filling the subarachnoid space with intracranial air (white arrow) and diffuse parenchymal swelling (white dotted arrow), mimicking a subarachnoid hemorrhage. **(B) **Subtractional magnetic resonance imaging showed no vascular malformation or intracranial hemorrhage. **(C) **The washed-out subarachnoid space after conservative management for one day (black arrow).

One day after the procedure, our patient complained of severe headache with sustained high blood pressure of over 190mmHg systolic and 110mmHg diastolic. But, neurological disorientation was not observed (Glasgow Coma Scale = 15). A CT of his brain showed a SAH-like appearance with intracranial air (Figure 3). Diffuse sulcal effacement, obliterated basal cisterns and hyperdensity in the interhemispheric fissure and perichiasmatic, perimesencephalic and sylvian cisterns were also suspected (Figure 3). A sequential angiography and subtractional MRI were performed, but no definite intracranial vascular malformation or intracranial hemorrhage was observed (Figure 3). A lumbar puncture showed normal opening pressure. Examination of his cerebrospinal fluid revealed the following: leukocytes 0 cells/mm^3^; erythrocytes 3 cells/mm^3^, without xanthochromia; and normal protein and glucose levels. His headache disappeared and no neurological aggravation was observed after conservative management. A follow-up CT scan after one day revealed no definite intracranial hemorrhage (Figure 3), and our patient was discharged with improved low back pain without neurological deficit. He remained neurologically asymptomatic with improved low back pain during three months of follow-up.

## Discussion

A SAH-like appearance in imaging has received only limited attention in the medical literature. In a previously reported case [5], the clinical presentation was compatible with spontaneous SAH and the CT images were interpreted as showing SAH with diffuse cerebral edema. The incidence of erroneous diagnoses of an apparent SAH has not been determined, but several radiographic mimics of SAH have been reported, including idiopathic intracranial hypertension, viral or pyogenic leptomeningitis, diffuse cerebral edema, pseudotumor cerebri, metabolic disorder, septic shock, CT appearance of intrathecally administered contrast material, and leakage of high-dose intravenous contrast medium into the subarachnoid spaces [3,5,7-10]. This above described situation has been alluded to, and increased density of the dura on CT mimicking a SAH has been reported in an autopsy study in patients who had increased intracranial pressure of non-SAH etiology [8]. But, the contrast enhancement of the subarachnoid space after an L-PEN has not previously reported in the literature.

To understand the etiology of a SAH-like appearance after L-PEN, we here review the modified procedure of L-PEN [11]. A specially designed RK^®^ needle (RK^®^ needle, Epimed International Inc.) was introduced into the sacral epidural space under fluoroscopy. Once the needle was confirmed to be in the epidural space, we carried out a lumbar epidurogram, using approximately 2 to 5mL of contrast. The filling defects were identified by examining the contrast flow into the nerve roots. Intravascular or subarachnoid placement of the needle or contrast was avoided; if such malpositioning occurred the needle was repositioned, as in this case (Figure 2). After appropriate determination of epidurography, a Racz^®^ catheter (Racz^®^ catheter, Epimed International Inc.), which is a spring guided reinforced catheter, was slowly passed through the needle to the area of the filling defect or the site of pathology determined by MRI, CT or patient symptoms. Following positioning of the catheter into the appropriate area, adhesiolysis was carried out by mechanical means and the injection of sodium chloride solution. Following completion of the injection, the catheter was taped using a bio-occlusive dressing, and the patient was turned to supine position and transferred to the recovery room. The catheter was flushed with normal saline and was removed and checked for intactness after a scheduled flushing for 1 or 2 days. The patient was ambulated if all parameters were satisfactory, intravenous access was removed and the patient was discharged home with appropriate instructions.

The contrast mimicking an SAH could have originated from the malposition of the neuroplasty catheter to the intradural space on the first attempt (Figure 2). The intracranial air that was shown in the brain CT scan with an SAH-like appearance could also have derived from the puncture of the dural sac (Figure 3). But, it is not well understood how the contrast in the lumbar subarachnoid space reached the intracranial cistern space. There is a considerable distance between the intracranial cistern and lumbar subarachnoid space, and the heavy contrast medium settles into lower lesions through gravity [12]. Indeed, experimental results indicate that contrast media are normally eliminated directly from the lumbar subarachnoid space into the vascular circulation without significant accumulation in the intracranial cisterns [13,14]. We believe that our patient’s position in the recovery room and his high blood pressure may have contributed to this rare complication after L-PEN.

During an L-PEN, the most important factor to prevent a similar complication is to avoid malpositioning the catheter, but it is always a risk. Therefore, we believe that the clinician should always be prepared for a similar complication, although it is not always as dangerous as in our case. An interesting study by Haughton et al. in 1982 [12] compared cisternal cerebrospinal fluid concentration in Macaque monkeys undergoing metrizamide myelography in sitting and recumbent positions. Serum iodine concentrations were lower when animals were kept supine than when kept sitting, and observed cisternal iodine concentrations were higher in supine animals than in the sitting ones. These results may indicate how to prevent similar complications of L-PEN. The authors recommended that, in the recovery room, the patient should be placed in a supine position with the head elevated and adequate fluid hydration and blood pressure control. As the results from the study by Haughton *et al.* show, this position and management are helpful for reducing the contrast concentration in the cranial cisterns even though the contrast was administered to the lumbar subarachnoid space.

## Conclusion

We have reported a rare case of imaging contrast mimicking a SAH after L-PEN. The physician should keep in mind a rare case like this, and placing the patient in a supine position with head elevation is necessary to avoid similar complications after L-PEN.

## Consent

Written informed consent was obtained from the patient for publication of this case report and any accompanying images. A copy of the written consent is available for review by the Editor-in-Chief of this journal.

## Abbreviations

CT: Computed tomography; L-PEN: Lumbar percutaneous epidural neuroplasty; MRI: Magnetic resonance imaging; SAH: Subarachnoid hemorrhage.

## Competing interests

The authors declare that they have no competing interests.

## Authors’ contributions

CHO and GYJ completed the literature review and wrote the manuscript. SDA was the neurosurgeon responsible for the medical care of the patient, and provided the images, and read and approved the manuscript. SHC read and edited the manuscript. All authors read and approved the final manuscript.
